# Sleep-like State in Pond Snails Leads to Enhanced Memory Formation

**DOI:** 10.3390/biology13050336

**Published:** 2024-05-11

**Authors:** Kengo Namiki, Junko Nakai, Ken Lukowiak, Etsuro Ito

**Affiliations:** 1 Department of Biology, Waseda University, Tokyo 162-8480, Japan; k-namiki38295k2r@ruri.waseda.jp (K.N.); kbc-jun5211@ruri.waseda.jp (J.N.); 2 Hotchkiss Brain Institute, University of Calgary, Calgary, AB T2N 4N1, Canada; lukowiak@ucalgary.ca

**Keywords:** sleep, memory, escape behavior, *Lymnaea*, sleep-like quiescent state

## Abstract

**Simple Summary:**

The importance of sleep in memory formation has been demonstrated primarily in mammals. Some invertebrates exhibit a sleep-like state. We investigated the relationship between sleep and memory consolidation in the pond snail *Lymnaea stagnalis*, using learning to suppress escape behavior. Learning to suppress escape was observed in snails that were allowed a sleep-like period between training and memory-test sessions; however, escape suppression was not observed in snails that were prevented from entering the sleep-like state between training and test sessions. The latency of the first escape was significantly shorter in the memory-test session in snails prevented from entering the sleep-like state between the training and memory-test sessions. Thus, the sleep-like state enhanced memory consolidation after escape behavior suppression learning in the snails. These data are consistent with the hypothesis that a sleep-like state plays an important role in memory formation in an invertebrate model system.

**Abstract:**

To test the hypothesis that a sleep-like quiescent state enhances memory consolidation in the pond snail *Lymnaea stagnalis*, we interposed a period in which snails experienced either a quiescent, sleeping state or an active, non-sleeping state following escape behavior suppression learning (EBSL). During EBSL training, the number of escapes made by a snail from a container was significantly suppressed using an external aversive stimulus (punishment). After training, the snails were divided into two groups. One group of snails was allowed to move freely and to experience a sleep-like quiescent state for 3 h in distilled water. The other group was stimulated with a sucrose solution every 10 min to keep them active (i.e., non-sleeping). In the memory test, escape behavior was suppressed in the group that experienced the quiescent state, whereas the suppression was not observed in snails that were kept active. Additionally, the latency of the first escape in the memory test was shorter in the snails kept active than in those that experienced the quiescent state. Together, these data are consistent with the hypothesis that a sleep-like quiescent state enhances EBSL memory consolidation in *L. stagnalis*.

## 1. Introduction

In mammals, sleep is important for the consolidation of learning into long-term memory [1]. For example, when using electro-encephalographic (EEG) criteria, sharp wave ripples, which represent a highly synchronized pattern of neuronal activity, are observed in the hippocampus during sleep [2]. These ripples are thought to modulate the activity of many brain regions, including the hippocampus, and play an important role in neuronal activity leading to memory formation in the hippocampus and other brain regions during sleep. In addition, associative memory consolidation is thought to occur during non-rapid eye movement sleep [3]. The question we ask here is whether behavioral changes associated with sleep lead to better memory formation in an invertebrate such as the pond snail *Lymnaea stagnalis* (Linnaeus, 1758).

To test our hypothesis, we must first describe how we define what sleep is in invertebrates. Because it is difficult to use EEG criteria in invertebrates, sleep is primarily defined behaviorally. Invertebrate sleep is defined as follows: (1) elevated arousal threshold; (2) reversible behavioral quiescence; (3) compensatory rebound sleep after sleep deprivation; (4) specific sleep posture; and (5) a preferred resting location [4,5,6,7,8,9,10]. In a related gastropod mollusk, *Aplysia californica*, sleep-like states have been systematically defined based on the above criteria and sleep-like resting states persisting for up to 100 min have been recorded during the night [11].

In *Drosophila melanogaster*, two research groups have reported a resting behavior with behavioral characteristics similar to sleep [12,13]. They defined sleep as occurring when flies were immobile for 1–25 min. Additionally, changes in sleep duration (i.e., immobility) were controlled by the circadian cycle, and sleep deprivation occurred when external stimuli were applied to the flies (i.e., to keep them mobile). Finally, responsiveness to external stimuli was lower in immobile individuals than in those who were active immediately before the stimuli were applied. These findings are consistent with the behavioral criteria for sleep in mammals. As a result, research in *D. melanogaster* has shown not only that sleep facilitates learning in wild-type and memory-impaired flies, but also that sleep deprivation impairs the acquisition and consolidation of new memories [14,15].

In the pond snail *L. stagnalis*, which has long been used to elucidate learning and memory mechanisms [16,17,18,19,20], Stephenson and Lewis (2011) characterized a quiescent state as a sleep-like state [21]. Snails spontaneously entered this quiescent state, characterized by postural relaxation of the foot, mantle, and tentacles, and the cessation of radula rasping [21]. This state was reversed (i.e., aroused) by both appetitive (e.g., sucrose solution) and aversive (e.g., tactile) stimuli. We followed these criteria to determine whether a snail was, or was not, in a sleep-like quiescent state.

Here, we took advantage of escape behavior suppression learning (EBSL) and the hypothesized relationship between memory consolidation and sleep in *L. stagnalis* [17,22,23,24]. In EBSL, snails attempt to escape from a container. However, when a KCl solution, which is an aversive stimulus (i.e., punishment), is placed outside the container and a snail touches it with its mouth, the number of escapes decreases. That is, the KCl stimulus suppresses the escape response. After successful training and memory consolidation, snails returned to the container exhibit a significantly longer latency before escaping from the container and make significantly fewer escape attempts. Thus, the total number of escapes and the latency of the first escape serve as an indicator of memory. Therefore, after training, we examined the changes in both the number of escapes and the latency to the first escape in both a sleep-like cohort and an active (i.e., non-sleep-like) cohort of snails.

## 2. Materials and Methods

### 2.1. Snails

*L. stagnalis* with a shell length of 18–25 mm were used. Snails were maintained in dechlorinated tap water under a 12 h light:12 h dark cycle at 21–25 °C and fed with Japanese mustard spinach (*Brassica rapa* var. *peruviridis*, known as Komatsuna in Japanese). The inbred laboratory strain of *L. stagnalis* used here was originally derived from stocks maintained at Vrije Universiteit Amsterdam.

### 2.2. Sleep-like Quiescent State

We examined how often and for how long snails exhibited a sleep-like quiescent state during a 3 h period. Two cohorts of naive snails were used. In the first group, each snail was placed on a 35 mm Petri dish lid containing distilled water (DW), and we measured the duration of the sleep-like quiescent state. In the second group, each snail was also placed onto a 35 mm Petri dish lid, but each snail was stimulated with a 10 mM sucrose solution for 15 s every 10 min (i.e., during the remaining 9 min and 45 s the snails were placed in DW). Therefore, snails in the second group were kept active and not allowed to enter the quiescent state (i.e., sleep). In both groups, we measured the duration of the quiescent period. We used a stopwatch and visually monitored the behavior.

### 2.3. Escape Behavior Suppression Learning

**Figure 1:**
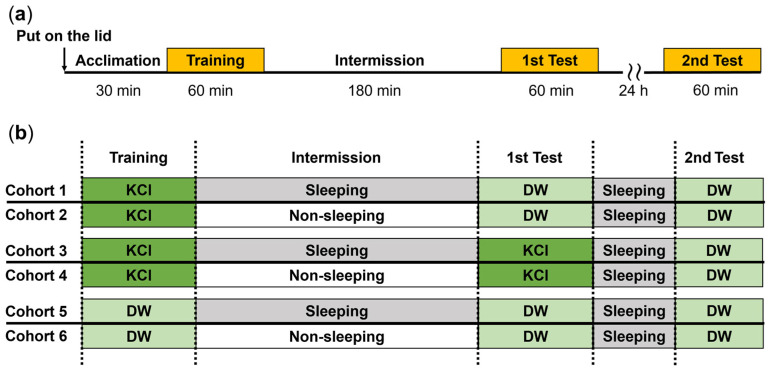
EBSL experiments with the 3 h interval (either quiescent or active state) between training and the first memory test: (**a**) timeline for training and 1st and 2nd memory test of EBSL; and (**b**) six cohorts of snails received EBSL training. In the training and test sessions, the external solution outside the Petri dish lid was either KCl or DW. The number of snails was 40 each in the training and the 1st Test (i.e., performance on the 1st day), and 20 each in the 2nd Test (i.e., performance on the 2nd day).

Escape behavior suppression learning (EBSL) experiments were conducted using the procedures described in previously published reports [17,22,24]. Snails were collected from their home aquaria and individually placed onto a 35 mm Petri dish lid filled with DW at a depth of 3 mm for 30 min to acclimate to DW (Figure 1). Then, the Petri dish lids were placed on a tray with either a 100 mM KCl solution (punishment) or just DW (control). First, there was a 60 min training period. The number of escapes (i.e., snails climbing over the Petri dish lid) was recorded (Training indicated in figures). The timing of the escape was defined as the moment when *L. stagnalis* leaned over the lid and put its head onto the tray containing either KCl or DW. Following the escape, the snail was moved back to the center of the Petri dish lid. In addition to recording the number of escapes, the latency of the first escape was also recorded. For individuals that did not escape during the 60 min training session, the first escape was considered to have a latency of 60 min. Next, we imposed either the sleep-like quiescent or the active (i.e., non-sleeping) period for 3 h. Previously, Nakai et al. (2020) used anisomycin (a protein synthesis inhibitor) and actinomycin (a transcription inhibitor) to show that long-term memory formation following a training procedure like that used here occurred about 2 h after the training procedure [25]. Therefore, the imposed 3 h interval following training was sufficient for long-term memory formation to occur. In the 3 h interval, snails were individually placed into a 50 mL tube containing 5 mL DW. In the sleep-like quiescent group, the DW was changed every 10 min. In the active group, we applied 10 mM sucrose for 15 s every 10 min. Between sucrose applications, the sucrose solution was replaced with DW. Exchanges of these solutions were performed using a 5 mL pipette. Finally, there was an initial memory test (1st Test in figures) given to all the snails for 60 min. The same procedure was used as in the training period.

To determine if memory for EBSL persisted longer than 3 h, both groups following the first test for memory, were subjected to another EBSL experiment 24 h later (2nd Test in figures). It should be noted that all the snails tested 24 h later were allowed to remain in the quiescent state for the 24 h before the second EBSL memory test (see Figure 1).

All the EBSL experiments were conducted between 8:30 a.m. and 2:30 p.m. Each group was experimented at the same clock time. The lights in the room were turned on at 7 a.m. and turned off at 7 p.m.

### 2.4. Statistics

Data were plotted in boxplot mode. Statistical analyses were performed by R (version 4.3.2) with a significance level of *p* < 0.05. A comparison was made between each two groups. The Wilcoxon rank sum test was used for paired comparisons (such as Training vs. 1st Test in figures), and the Wilcoxon signed rank sum test was used for unpaired comparisons (such as Training vs. 2nd Test in figures). Finally, *p* values for multiple comparisons were adjusted using the Holm method.

## 3. Results

### 3.1. Exhibition of Sleep-like Quiescent State for 3 h Period

**Figure 2:**
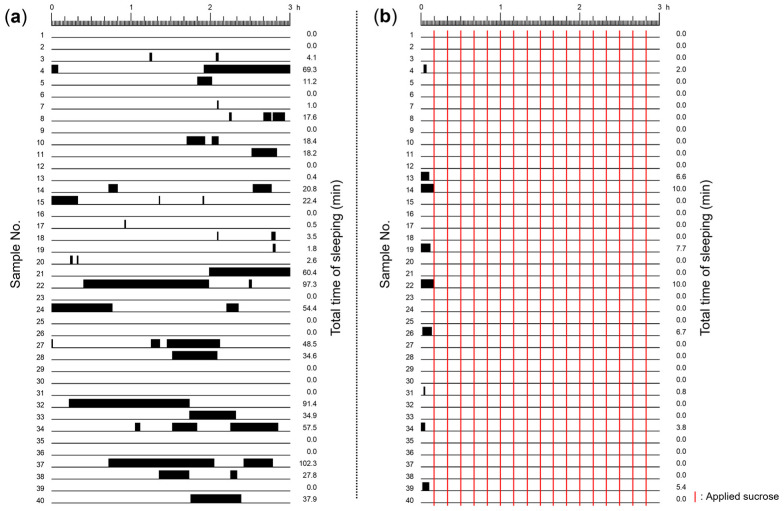
Raster representation of the sleep-like quiescent state exhibited in the 3 h periods: (**a**) snails were exposed to only DW, and experienced sleep-like quiescent states (black bars); and (**b**) snails were stimulated with a sucrose solution every 10 min to keep them active (during the remaining 9 min and 45 s the snails were placed in DW) (see red lines). The number on the left indicates the individual identification number of the snail, and the number on the right indicates the total sleep-like quiescent state time in min.

We first determined how often, and for how long, snails exhibited the sleep-like quiescent state or the non-sleeping active state during a 3 h period (Figure 2). Snails in only DW for 3 h exhibited sleep-like quiescent states (i.e., little or no movement) (Figure 2a). On the other hand, snails in DW that were stimulated with sucrose exhibited a sleep-like quiescent state in the initial 10 min period (i.e., before the sucrose stimulation); when sucrose stimulation started snails did not exhibit the sleep-like quiescent states (Figure 2b). Thus, frequent stimulation with the sucrose solution kept snails active (i.e., non-sleeping).

### 3.2. Comparison of Escape Behavior after EBSL between Snails That Experienced the Sleep-like Quiescent State and Those in the Active State

In the next series of experiments, we used 6 cohorts of naive snails. Following EBSL training, snails were assigned to different combinations of the sleep-like quiescent state or the non-sleeping active state during the 3 h intermission between training and the 1st memory test. Thus, the different cohorts of snails (see below) encountered 100 mM KCl (punishment) or DW (control) when they climbed over the Petri dish lid.

**Figure 3:**
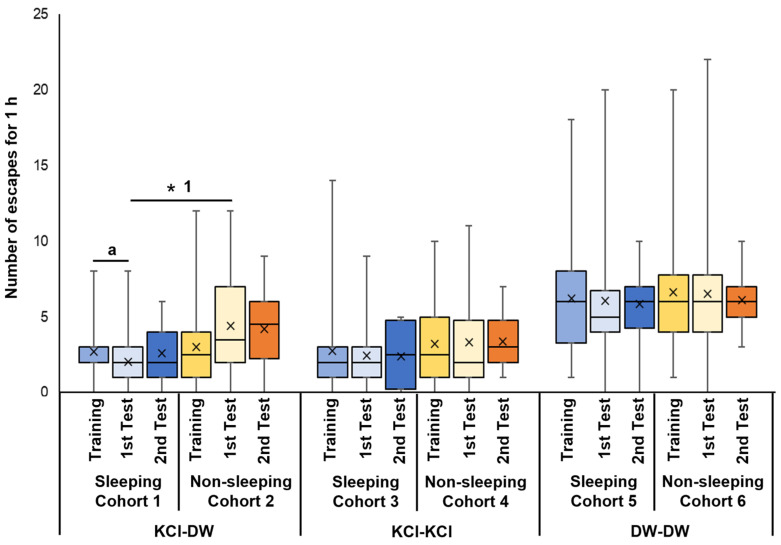
Number of escapes in training and memory-test periods of EBSL. Blue boxes indicate the data from the training period before the experience of sleep-like quiescent state. Light blue boxes indicate the data from the 1st memory-test period after the experience of sleep-like quiescent state. Dark blue boxes indicate the data from the 2nd memory-test period 24 h later. Yellow boxes indicate the data from the training period before the experience of non-sleeping active state. Light yellow boxes indicate the data from the 1st memory-test period after the experience of non-sleeping active state. Orange boxes indicate the data from the 2nd memory-test period 24 h later. The left 6 bars show the data obtained from cohorts subjected to KCl in the training period and DW in the 1st and 2nd memory periods. The middle 6 bars show the data obtained from the cohorts subjected to KCl in the training period and KCl in the 1st and 2nd memory-test periods. The right 6 bars show the data obtained from the cohorts subjected to DW in the training period and DW in the 1st and 2nd memory-test periods. ‘a’ indicates *p* = 0.032, N = 40; ‘* 1’ indicates *p* = 0.019, N = 40.

Cohort 1 snails were trained with KCl, then experienced the sleep-like quiescent state in the 3 h period between the training and memory-test sessions. The first memory-test session occurred 3 h after training while the second memory-test session occurred 24 h after training. In this cohort, there was a significant decrease in the number of escapes between the training (Training) and the first memory test (1st Test) sessions (*p* = 0.032 from the Wilcoxon rank sum test, N = 40; see ‘a’ for the 1st and 2nd bars from the left in Figure 3). This result showed that the decrease in escape behavior seen in the memory test indicated that memory had formed following the training session. However, a 24 h memory was not observed (see 2nd Test (N = 20)).

Cohort 2 snails were trained with KCl, experienced the 3 h active state before the first memory test, and were tested for memory in DW. There were no significant differences in the number of escapes between the training (Training, see the 4th bar in Figure 3) and the memory-test sessions (1st Test and 2nd Test, see the 5th and 6th bars in Figure 3). This result is consistent with the hypothesis that the lack of sleep hindered memory formation.

When Cohort 1 and Cohort 2 were compared and the *p* value was adjusted by multiple comparisons (i.e., Holm method), a significant difference was observed between the 3 h memory test in the two cohorts (i.e., 2nd bar and the 5th bar) (*p* = 0.019, see ‘* 1’ in Figure 3). Thus, the data indicate that memory formation was suppressed in snails that were not allowed to sleep.

Cohort 3 snails were trained with KCl, experienced the 3 h sleep-like quiescent state, and were tested for memory in KCl. There were no significant differences in the number of escapes between the training, the 1st memory-test period, and the 2nd memory-test periods (Figure 3). That is, when memory was tested using the KCl solution, snails did not show a significant difference in the number of escapes between the training and memory-test sessions.

Cohort 4 snails were trained with KCl, experienced the 3 h active state, and were tested for memory in KCl. There were no significant differences in the number of escapes between the training, the 1st memory-test period and the 2nd memory-test period (Figure 3). Thus, as with the cohort 3 snails, the cohort 4 snails did not show a significant difference in the number of escapes between the training and memory-test sessions.

Cohort 5 snails were trained with DW, experienced the 3 h sleep-like quiescent state, and were tested for memory in DW. There were no significant differences in the number of escapes between the training, the 1st memory-test period and the 2nd memory-test period (Figure 3). These data show that training in DW was not sufficient for EBSL learning or memory formation.

Finally, Cohort 6 snails were trained with DW, experienced the active state, and were tested for memory in DW. Again, there were no significant differences in the number of escapes between the training, the 1st memory-test period, and the 2nd memory-test period (Figure 3). Thus, this cohort of snails behaved in a similar manner to the Cohort 5 snails.

Therefore, when the snails were EBSL trained with KCl, kept in a sleep-like quiescent state and then tested for memory in DW, the escape behavior in the memory test was significantly suppressed compared to that shown in the training session. Thus, a memory was formed. On the other hand, when the snails were subjected to the stimuli to keep them active after the EBSL training, the escape behavior was not suppressed, and the memory was not formed.

### 3.3. Comparison of Latency of the 1st Escape Behavior after EBSL between Snails That Experienced the Sleep-like Quiescent State and Those in the Active State

**Figure 4:**
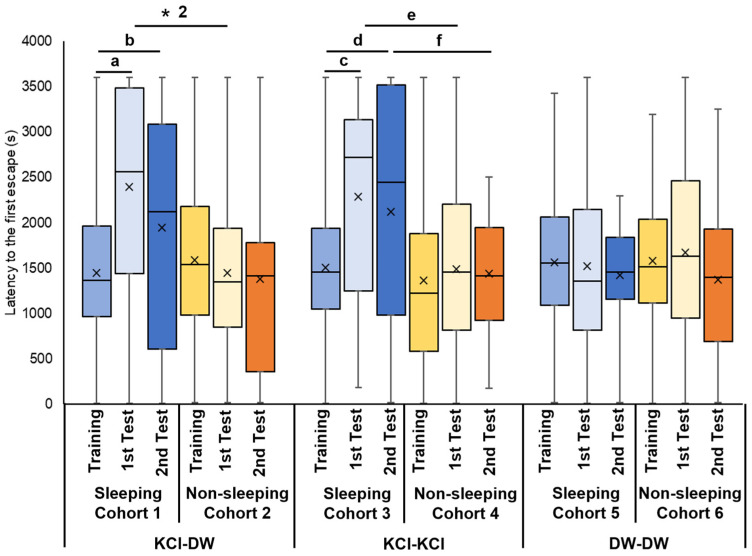
First escape latency in EBSL training and memory-test periods. Blue boxes indicate the data from the training period before the experience of sleep-like quiescent state. Light blue boxes indicate the data from the 3 h memory-test (i.e., 1st memory-test) period after the experience of sleep-like quiescent state. Dark blue boxes indicate the data from the 24 h memory test (i.e., the 2nd memory test). Yellow boxes indicate the data from the training period before the experience of a non-sleeping active state. Light yellow boxes indicate the data from the 3 h memory-test (i.e., 1st memory-test) period after the experience of non-sleeping active state. Orange boxes indicate the data from the 24 h memory test (i.e., the 2nd memory test). The left 6 bars show the data obtained from cohorts subjected to KCl in the training period and DW in 1st and 2nd memory-test periods. The middle 6 bars show the data obtained from the cohorts subjected to KCl in the training period and KCl in the 3 h and 24 h memory-test periods (i.e., 1st and 2nd memory tests). The right 6 bars show the data obtained from the cohorts subjected to DW in the training period and DW in the 3 h and 24 h memory-test periods (i.e., 1st and 2nd memory-test periods). ‘a’ indicates *p* < 0.001, N = 40; ‘b’ indicates *p* = 0.035, N = 40 for the 1st bar and 20 for the 3rd bar; ‘c’ indicates *p* = 0.006, N = 40; ‘d’ indicates *p* = 0.007, N = 40 for the 7th bar and 20 for the 9th bar; ‘e’ indicates *p* = 0.046, N = 40; ‘f’ indicates *p* = 0.009, N = 20; ‘* 2’ indicates *p* = 0.041, N = 40.

We also recorded the first escape latency in all 6 cohorts (Figure 4). In Cohort 1, snails were trained with KCl, experienced the sleep-like quiescent state, and were tested in DW. The 1st escape latency in both the 3 h and 24 h memory tests (i.e., 1st memory test and 2nd memory tests) were significantly prolonged compared the latency in the training session (*p* < 0.001 for ‘a’ from the Wilcoxon rank sum test and *p* = 0.035 for ‘b’ from the Wilcoxon signed rank sum test; Figure 4). Thus, snails in this cohort showed an increased hesitation to escape in the memory tests following the KCl training session and the 3 h quiescent state.

In Cohort 2 (i.e., snails were trained with KCl, experienced the active state, and were tested in DW), there were no significant differences in the first escape latency between the training, the 3 h and 24 h memory tests (i.e., 1st and the 2nd memory tests (Figure 4)).

When Cohort 1 and Cohort 2 were compared and the *p* value was adjusted by multiple comparisons, a significant difference was observed between the 3 h memory tests in the respective cohort (*p* = 0.041 adjusted by the Holm method, see ‘* 2’ in Figure 4). In the 3 h memory test, between snails that were allowed to sleep and snails that were not allowed to sleep, the latency for the first escape for significantly longer in snails allowed to sleep.

Cohort 3 snails that were trained with KCl, experienced the sleep-like quiescent state, and were tested for memory in KCl, the 1st escape latency in the 3 h memory test (i.e., 1st memory test) and the 24 h memory test (i.e., 2nd memory test) periods was significantly prolonged compared to the training session (*p* = 0.006 for ‘c’ from the Wilcoxon rank sum test and *p* = 0.007 for ‘d’ from the Wilcoxon signed rank sum test; Figure 4). This result showed that there was a significant increase in latency (i.e., ‘hesitation’) in snails that were trained with KCl stimulation and allowed to sleep.

Cohort 4 snails were trained with KCl, experienced the active state, and were tested for memory in KCl. There were no significant differences in the 3 h memory-test period (i.e., 1st memory-test) and the 24 h memory-test period (i.e., 2nd memory-test) between the training and test sessions (Figure 4). The comparison between Cohorts 3 and 4 highlighted the hesitation in snails that were trained with KCl stimulation and allowed to sleep (Figure 4; *p* = 0.046 for ‘e’ from the Wilcoxon signed rank sum test and *p* = 0.009 for ‘f’ from the Wilcoxon signed rank sum test).

In Cohort 5 snails that were trained with DW, experienced the sleep-like quiescent state, and were tested for memory in DW, there were no significant differences in the first escape latency between the training and the subsequent (3 h and 24 h) memory tests (Figure 4).

Finally, in Cohort 6 snails that were trained with DW, experienced the active state, and were tested for memory in DW, there were no significant differences in the first escape latency between the training and the subsequent (3 h and 24 h) memory tests (Figure 4).

The first latency escape data showed that snails that experienced the sleep-like quiescent state after the training session, combined with KCl punishment, exhibited a longer first latency escape in the memory test compared to the training test. That is, these snails were hesitant to escape from the Petri dish lid during the memory test. On the other hand, when the snails experienced the active state after EBSL training, there was no significant increase in the first escape latency, suggesting that memory had not formed.

## 4. Discussion

Escape suppression by EBSL training was observed in *L. stagnalis* that were allowed to have a sleep-like quiescent period between the training and memory-test sessions; however, escape suppression was not observed in snails that experienced the non-sleeping active period between the training and test sessions. Consistent with those findings are the first escape latency data. Thus, the first escape latency in the memory test was significantly shorter in the snails experiencing the active vs. the quiescent interval between the training and memory-test sessions. We conclude that the sleep-like quiescent state enhances memory formation (i.e., consolidation) when tested 3 h and 24 h after training. This was especially seen when we compared the first escape latencies. That is, giving snails a 3 h period of quiescence after training resulted in an increase in hesitation to leave the Petri dish even 24 h later.

Previously, the relationship between a sleep-like quiescent period and memory consolidation was explored in another mollusc, *A. californica*. A sleep-like state was shown to be essential for memory consolidation as a form of inhibitory operant conditioning [26,27,28]. With our *L. stagnalis* model system, we will investigate in the future whether there is a positive role for a sleep-like state for memory formation in a classical conditioning procedure (e.g., CTA) [29]. Previously, Wagatsuma and colleagues showed that CTA training in the morning in *L. stagnalis*, possibly as a result of a circadian rhythm influence, results in better learning and memory formation than when snails are trained in the afternoon [30]. In addition, we would like to note that the data in the Stephenson and Lewis study demonstrated that *L. stagnalis* exhibit a sleep-like quiescent state even during the “light phase” of the circadian rhythm [21].

It has been demonstrated in *L. stagnalis* that memory formation following training undergoes different phases (see Figure 8 in Marra et al., 2013) [31]. In addition, memory formation can also be parsed into short (minutes), intermediate (up to 3 h) and long term (greater than 3 h), based on behavioral and RNA synthesis data [32]. It has also been shown that these memories differ in their physiological mechanisms. In a previous study in *L. stagnalis* using the CTA training method, long-term memories were formed at least two hours after training [25]. Thus, knowing these time windows, we hypothesized that there would be differences in memory formation in snails kept active (no memory) compared to those allowed a quiescent period.

In *D. melanogaster*, the importance of sleep in long-term memory formation has been indicated in classical aversive olfactory aversion learning. Specifically, memory formation was significantly impaired by the deprivation of sleep 4 h after training, compared to sleep deprivation before training [33]. It has also been reported that in *A. californica*, short (4 h) or chronic (2 nights, 6 h) sleep deprivation before learning training interferes with the formation of long-term and short-term memories [26,27]. We have, as of yet, not determined whether sleep deprivation before any sort of associative learning training (e.g., operant or classical conditioning) will have a significant impact on the snails’ ability to learn or form memory. Such experiments are now being planned for *L. stagnalis*.

The new experiments discussed here suggest that a sleep-like quiescent interval between training and memory-test sessions enhances memory formation in snails, in the same manner that it does in mammals. In *L. stagnalis*, upregulation of the expression of cAMP-response element binding protein 1 (CREB1), CREB2, CREB-binding protein, and monoamine oxidase has been shown after EBSL, and we believe that it is likely that the upregulation of CREB and its related proteins does not occur when snails are kept in the active state between sessions [34,35]. We will perform experiments in the near future to determine if this is a correct assumption.

## 5. Conclusions

In invertebrates, sleep is defined behaviorally as a quiescent state. We were able to show that a sleep-like state promoted memory formation, whereas an active state retarded memory formation. In addition, the use of a KCl stimulus in EBSL training rather than a DW stimulus caused learning to be formed that was subsequently consolidated into a long-term memory. In the memory test, escape behavior was suppressed in the snails that experienced the quiescent state, whereas the suppression was not observed in snails that were kept active. The latency of the first escape in the memory test was shorter in the snails kept active than in those that experienced the quiescent state. The present study therefore adds to our understanding that sleep is also important for memory consolidation in invertebrates such as *L. stagnalis*.

## Data Availability

All data that support the findings of this study are available from the corresponding authors upon reasonable request.
